# The Conditions of Successful Telework: Exploring the Role of Telepressure

**DOI:** 10.3390/ijerph191710634

**Published:** 2022-08-26

**Authors:** Junyoung Hong, Steve Jex

**Affiliations:** Department of Psychology, University of Central Florida, Orlando, FL 32816, USA

**Keywords:** telework, work–life conflict, telepressure

## Abstract

The purpose of this paper is to explore the causes of the inconsistent relationship between telework and work–life conflict, which has been reported in the research literature. We predicted that the qualitative aspects of telework, direction of work–life conflict, and telepressure would influence whether telework decreases work–life conflict. To test these predictions, data from a sample of 328 workers enrolled in the online subject recruitment platform, Prolific, were collected three times, with a one-month interval between each data collection. The analysis, based on these data, revealed that the qualitative aspects of telework had no impact on the relationship between telework and work–life conflict. In addition, telework was significantly related only to work-to-life conflict, but not life-to-work conflict. Finally, the moderating effect of telepressure was significant, such that the positive impact of telework on work–life conflict was found only for people reporting low telepressure. Based on the research findings, theoretical and practical implications were discussed.

## 1. Introduction

Due to the COVID-19 pandemic, the world has been undergoing significant social changes. Among these many changes, one of the most widespread has been a change in the physical location of the workplace, defined as *telework*. Although telework has drawn the attention of researchers, due to the recent COVID-19 pandemic, it has been implemented since it was proposed, almost four decades ago, as a means of enhancing work flexibility [1,2]. Given this timeframe, some experts have said that the COVID-19 pandemic, at least with respect to telework, has just accelerated the social changes that were already underway, rather than causing those social changes [3,4]. Furthermore, many people have predicted that the shift to telework will be maintained even after the Coronavirus subsides [3,4,5]. However, even though this change has already arrived in our society, it is unclear whether the impact of telework is consistently positive. One argument supporting the potential positive effects of telework is based on its impact on the work–nonwork interface. Many studies have examined the impact of telework on work–family conflict, but the results have been so mixed that it is unclear whether telework is positive or negative [1,6,7,8,9]. Given that one of the fundamental purposes of telework is to increase the autonomy of employees, and there are expectations that this autonomy will alleviate work–family conflict [1], these mixed findings suggest the need for more research on the relationship between telework and work–nonwork conflict.

The present study aims to explore several factors that may affect the relationship between telework and work–life conflict. We focused on work–life conflict, instead of work–family conflict, because work–life conflict covers a more general and wider area than work–family conflict. For a deeper understanding of the relationship between telework and work–life conflict, we explored whether specific qualitative attributes of telework, as well as the direction of work–life conflict, impact this relationship. With regard to the qualitative attributes of telework, the motivation to participate in telework and regularity of telework were considered. Regarding the direction of work–life conflict, it was expected that the effects of telework would differ depending on whether it was work interfering with other life domains or other life domains interfering with work.

The present study also examined whether another psychological attribute, telepressure, could impact the relationship between telework and work–life conflict. Telepressure is defined as employees’ perceived pressure to respond quickly to job-related messages delivered through information and communication technologies (ICTs [10]). As ICTs have been developed rapidly and many technologies are frequently used at work, this has led to a problem labeled “over-availability syndrome”. This syndrome refers to the idea that some teleworkers feel like they must respond promptly to every form of electronic communication during working hours, even if these communications do not require an urgent response [11]. Therefore, we argue that the concept of telepressure could provide a possible explanation for the inconsistent effects of telework, especially the negative effects. Accordingly, this study examined whether telepressure moderates the relationship between telework and work–life conflict.

In summary, the goal of this study was to explore the reasons why telework sometimes does not alleviate work–life conflict, even though this was one of the original justifications for telework arrangements. In the following sections, theories and previous research findings related to the current study variables are discussed; based on this review, a series of research hypotheses are then presented.

### 1.1. The Impact of Telework on Work–Life Conflict

Telework is defined as a system or work arrangement where individuals can perform their work in a place they choose, rather than in a central workplace. The initial purpose of telework was to reduce the costs of real estate and transportation and increase employee autonomy to help balance work and family duties [1,7]. Since telework is a system implemented by organizations, previous studies examining telework have focused largely on the consequences of organizational telework policies. Although telework has been implemented with several positive expectations, not all studies have found positive effects of telework. Indeed, regarding the impact of telework on work–nonwork conflict, some studies have shown that telework decreases work–life conflict [1], while other studies have shown the opposite effect [12,13]. However, even though the research findings have been mixed, considering that telework is deeply related to the management of boundaries between work and nonwork domains, those mixed results are not surprising. Boundary management is a concept derived from boundary theory, and the core idea of this theory is that people divide their lives into work and nonwork domains [14,15,16], and the clarity of the boundary varies from situation-to-situation and person-to-person. For example, one person may seek complete segmentation and create a strong boundary between work and life domains, while another may prefer to integrate the two domains, creating a relatively blurred boundary. When viewed from this perspective, telework has been considered a system that can increase flexibility and permeability of the boundary between the work and nonwork domains because teleworkers typically handle most of their work tasks outside of the workplace [1,17]. What is important here is that one cannot say whether a flexible boundary is always better than a strong boundary. Indeed, Clark [18] argued that role blending, due to flexible boundaries, can help individuals to make more skilled role transitions across two different life domains; however, at the same time, it can also cause role blurring or conflict. Likewise, many previous studies have shown inconsistent effects of telework on the interface between work–nonwork domains; for example, some studies have found that telework can alleviate work–family conflict, while others have also reported that telework can increase work–family conflict [1,8,12].

Work–family conflict, which has been frequently studied as an outcome of telework, refers to a state in which an individual has difficulty performing work and family roles simultaneously [19]. This concept of work–family conflict can be explained by the conservation of resource theory (COR theory [20]). The COR theory assumes that an individual’s total resources are limited, as well as that an individual tries to secure and maintain resources [20]. In addition, Hobfoll [20] stated that, if an individual perceives potential resource loss or loses resources, he or she will feel a high level of stress. Since it deals with stress caused by resource depletion, COR theory provides a useful explanation for why work–family conflict is stressful [21]. For example, if an individual invests most of his or her energy in a role in one domain, this can lead to role conflict between two domains. From these two theories, boundary and COR theory, we can infer both positive and negative effects of telework, and these conflicting effects have already been reported in several studies [1,6,7,8,9]. Therefore, the current study focused not only on examining the relationship itself, but also on exploring the factors that can affect this relationship. Moreover, unlike previous studies, we focused on work–life conflict, instead of work–family conflict, as an outcome variable of telework. Considering the recent demographic trends, in which single-person households already account for about 30% of the total population and their proportion is constantly increasing [22,23], studying the broader concept of work–life conflict, which covers a person’s overall private life (e.g., personal and family activity), provide more insights for research on the work–nonwork interface. Indeed, regarding research on the work–nonwork interface, there has been a call to explain the nonwork area beyond the family domain [24,25]. Accordingly, the research model adopted in the current study explored several factors impacting the relationship between telework and work–life conflict.

What could potentially affect the relationship between telework and work–life conflict? In this study, we first considered the qualitative aspects of telework and direction of work–life conflict. The concept of the qualitative attributes of telework was derived from the idea that telework is not performed the same way by everyone. Many studies have focused on the quantitative aspects of telework (e.g., frequency or working hours) when measuring telework [1,6,9]; however, even if employees spend the same amount of time working from home, the effectiveness of telework may vary, depending on the individual’s motivation or characteristics of the telework system (e.g., regularity). For example, consider the difference between voluntary and involuntary telework. According to self-determination theory [26], autonomy, along with competence and relatedness, is one of the basic psychological needs that an individual requires to achieve well-being. In line with this argument, when an individual performs a task with high autonomy, positive outcomes are generally found, not only at work, but also outside of work [27,28,29]. Specifically, individuals performing tasks with high autonomy have reported a high level of job satisfaction, as well as higher psychological well-being and work–life balance. On the other hand, the consequences of low autonomy, or involuntary tasks, also need to be considered. Lapierre and colleagues [12] reported that involuntary telework, that is, telework that is mandated by organizational policies, could increase employee’s work–family conflict. Additionally, considering the current circumstances in which many organizations have been forced to require their employees to work from home, due to COVID-19, it is worth investigating the impact of individuals’ motivation to participate in telework. Regarding the specific effect of individual motivation, related to participation in telework, the present study speculated that individual motivation could moderate the relationship between telework and work–life conflict. More specifically, it was hypothesized that voluntary telework can alleviate work–life conflict, but involuntary telework would not be helpful in reducing work–life conflict.

**Hypothesis** **1.** *The voluntary nature of telework will moderate the relationship between telework and work–life conflict. Specifically, a negative (−) relationship is expected for voluntary telework, but it is not expected for involuntary telework.*

Another attribute of telework that may impact the relationship between telework and work–life conflict is the regularity of such work arrangements. If telework is performed regularly, it is more likely to alleviate role conflict, as intended. However, if telework is performed irregularly, this irregular work schedule may, rather, increase role conflict. Aguilera and colleagues [30] argued that most studies focusing largely on formal or regular forms of telework were a clear limitation of telework research because informal or irregular telework could result in different effects. Indeed, in previous research, the presence of irregular work shifts was considered one of the predictors of job burnout, along with role conflict [31]; in the same study, it was also reported that having irregular work shifts and job burnout were significantly correlated (r=0.53, p < 0.001). The results of this previous study suggest that the regularity of the work schedule should be considered when investigating role conflict. Accordingly, in this study, it was expected that the regularity of telework could moderate the relationship between telework and work–life conflict, and the corresponding hypothesis was derived.

**Hypothesis** **2.** *Regularity of telework will moderate the relationship between telework and work–life conflict. Specifically, a negative (−) relationship is expected for regular telework, but not for irregular telework.*

Next, the present study examined whether the direction of work–life conflict is an important factor impacting the relationship between telework and work–life conflict. Referring to previous studies discussing the direction of work–family conflict [32], work–life conflict can be divided into work-to-life conflict (WTLC) and life-to-work conflict (LTWC). Here, WTLC refers to the situation caused by work roles impeding life roles, and LTWC indicates the situation caused by life roles hindering work roles. Golden and colleagues [9] argued that the existing telework literature has generally ignored the direction of work–life conflict; therefore, the research findings could be conceptually and empirically limited. In other words, the two different directions might be one of the reasons why previous studies have reported mixed effects of telework on the work–nonwork interface. For example, in the case of parents with young children, WTLC may decrease while working from home, but LTWC may increase because they have no choice but to pay attention to their young children while working from home. Unlike WTLC, which telework has consistently been shown to reduce, it has been reported that LTWC is not significantly related to telework [6], or it is even positively related [9]. In terms of the reason why telework can increase LTWC, researchers have argued that staying outside of the workplace, due to telework, may lead to direct exposure to non-work demands and stronger pressure to perform them [9]. In sum, it has been shown that the relationship between telework and work–life conflict varies, depending on the direction of work–life conflict, with different explanations for this difference. Based on the previous research findings, the following two different relationships were hypothesized.

**Hypothesis** **3-1.** *Telework will be negatively associated with work-to-life conflict (WTLC).*

**Hypothesis** **3-2.** *Telework will be unrelated to life-to-work conflict (LTWC).*

### 1.2. The Role of Telepressure

**Figure 1:**
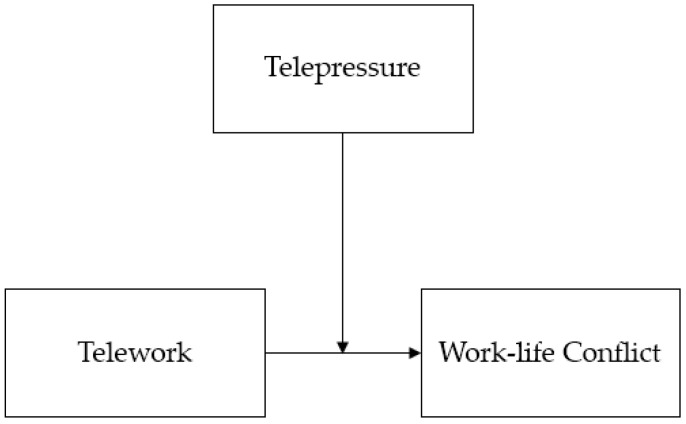
Research Model. *Note*. Within-person level interaction effect was estimated.

In addition to the attributes of telework and direction of work–life conflict, the present study also explores how telepressure impacts the relationship between telework and work–life conflict. Telepressure was a concept proposed along with the development of information and communication technologies (ICTs), and it is defined as an obsession with responding quickly to organizational communication using ICTs [10]. The irony of telepressure is that, even though ICTs were designed to enhance work flexibility and convenience in the communication between employees, the perceived flexibility and convenience may be reduced by telepressure [10]. Several studies have noted the potential dangers of this paradox and examined the impact of telepressure on individual well-being [33,34]. For example, it was found that a high level of telepressure led to exhaustion and sleep problems; moreover, telepressure decreased satisfaction, regarding work–life balance [33,34]. Acknowledging the negative effects of telepressure, some scholars have looked at factors that could increase telepressure [10,35]. First, Barber and Santuzzi [10] interpreted telepressure as an internalized perception and viewed it as a comprehensive result of the environmental factors increasing ICT demands and internal factors, such as personality traits. On the other hand, although Grawitch and colleagues [35] agreed that both environmental and internal factors should be considered as predictors of telepressure, their statistical analysis revealed that individual differences, especially maladaptive tendencies, such as workaholism and neuroticism, were stronger predictors than environmental factors, when it comes to explaining the variance of telepressure. Putting together these researchers’ arguments and research findings, telepressure can be seen as a variable that can change, depending on each person’s interpretation of the environment and different personality traits. In the present study, telepressure is proposed as a moderator of the relationship between telework and work–life conflict. Individuals with low telepressure may enjoy the benefits of telework, and their work–life conflicts can, therefore, be decreased. In contrast, individuals with high telepressure are more likely to experience the negative effects of telework, namely “over-availability syndrome”, which was described earlier. Accordingly, the following hypothesis was proposed, and the research model was presented in Figure 1.

**Hypothesis** **4.** *Telepressure will moderate the relationship between telework and work–life conflict. Specifically, a negative (−) relationship will be reported for those with low telepressure, but not for those with high telepressure.*

## 2. Materials and Methods

### 2.1. Participants and Procedure

A sample of 328 full-time workers holding various jobs (e.g., managerial, clerical, service/sales, production, IT/technical, etc.) were recruited via the online subject recruitment platform, Prolific. Considering the purpose of the present study, which is to investigate the inconsistent effects of telework, the population of this study was working adults who are engaging in telework. Therefore, the inclusion criteria were: (1) currently residing in the United States, (2) working full-time, and (3) performing telework at least one day per week. Participants ranged in age from at least 20 to at most 78 years old (*M* = 36.71, *SD* = 10.81). In the case of gender, the technology of demographic balancing provided by Prolific was used, so it was possible to secure an equal number of participants for males and females. More than half of participants reported that their level of education was higher than college graduation (“Less than a high school diploma” = 0%, “High school graduates, no college” = 5.2%, “Some college, no degree” = 9.1%, “Associate degree” = 7.3%, “Bachelor’s degree only” = 49.7%, and “Advanced degree” = 28.7%), and the current occupations were relatively diverse (“Managerial” = 29.6%, “Clerical” = 9.1%, “Service/Sales” = 12.2%, “Production” = 2.4%, “IT/Technical” = 22.9%, “Research” = 6.7%, and “Others” = 17.1%). Lastly, for the question asking about the number of children, 62.2% of respondents said they had no children, 12.8% had one child, 17.4% had two children, and 7.6% had three or more children.

The present study consisted of three measurement occasions. Reviewing previous studies examining the effects of telework, especially those using repeated measurements, it was found that various time intervals were applied, ranging from daily to two months [12,36]. Thus, it is not yet clear whether the effect of telework is immediate or requires a relatively long time. Considering that there is no specific guideline for setting the length of the time interval, this study applied a one-month interval. A one-month interval, which can be considered a middle value, was adopted because the within-person level variance of the study variables could be too small if the interval was too short, and the final response rate could be too low if the interval was too long. Among 4058 eligible samples for the target population of the present study in Prolific, we collected the responses from 328 people who responded to the first survey (14 January 2022), and 300 among them responded to the second survey (11 February 2022). The third survey (11 March 2022) was sent not only to the 300 people who answered the second survey, but also to all 328 people who answered the first survey, and 285 people among them responded. Therefore, the response rates for the second survey and third survey were 91% and 87%, respectively. Since the inclusion criteria for the present study were applied only to the first survey, the second and third surveys could include those who were not engaged in telework. Another important note in this study is that, since multilevel analysis was conducted, the data were used for the analysis, without getting rid of the case if a response was submitted to at least one of the three surveys. Regarding the measured variables for each survey, the demographics and variables in this study were measured in the initial survey; after that, only variables at the within-person level (e.g., telework, work–life conflict, and telepressure) were repeatedly measured.

### 2.2. Measures

#### 2.2.1. Telework

This study measured the extent of telework by using working hours. Specifically, total working hours per week and telework hours per week were asked, and then proportion scores (i.e., telework hours per week/total working hours per week) were calculated. This method of measuring the extent of telework in this way has been used in several studies [9,37].

#### 2.2.2. Qualitative Aspects of Telework

The present study also measured whether telework was voluntary, as well as whether it occurred on a regular basis. In one previous study, examining whether telework was voluntary, an individual’s perception of voluntariness of telework was measured using a Likert-type response scale [38]. In the case of regularity of telework, this was measured by how frequently employees engaged in telework [30]. In the current study, Likert-type response scales were adopted to measure whether telework was voluntary, as well as the regularity of telework. Specifically, both were measured by single-item questions with a 5-point Likert-type response scale (1 = *strongly disagree*, 5 = *strongly agree*). The question items are as follows: “I voluntarily participated in telework” and “My telework is carried out regularly (e.g., every day, every Friday, or every morning)”.

#### 2.2.3. Work–Life Conflict

To measure work–life conflict, 12 items, developed by Carlson and colleagues [32], were adapted. This scale was originally developed to measure work–family conflict, but several previous researchers have utilized this scale to assess work–life conflict [39,40]. In terms of the adaptation process, it was usually achieved by revising the “family” term to a more general term covering overall private life. The present study, followed the same procedure and average internal consistency (i.e., Cronbach’s α) across three data collections, was reported 0.93. Additionally, this measurement includes two directions, both work-to-life and life-to-work conflicts, and Cronbach’s α of each subdimension were 0.94 and 0.91, respectively. Lastly, the measurement utilized a 5-point Likert-type response scale (1 = *strongly disagree*, 5 = *strongly agree*), and example items are as follows: “My work keeps me from my personal and family activities more than I would like” (WTLC), and “Tension and anxiety from outside of work often weakens my ability to do my job” (LTWC).

#### 2.2.4. Telepressure

Perceived telepressure was assessed using six items developed by Barber and Santuzzi [10]. This scale was measured on a 5-point Likert-type response scale (1 = *strongly disagree*, 5 = *strongly agree*), and the average Cronbach’s α in this study was 0.93. Example items are as follows: “It’s hard for me to focus on other things when I receive a message from someone”, and “I feel a strong need to respond to others immediately”.

#### 2.2.5. Individual Differences

In the present study, several individual difference variables were also measured along with the research variables. Although details will be described later, it reflects the argument of Grawitch and colleagues [35], i.e., that the role of individual difference variables needs to be considered in the process of verifying the influence of telepressure on well-being-related outcomes.

- Personality Traits. To measure neuroticism, conscientiousness, and agreeableness, this study utilized 30 items from the International Personality Item Pool (Big-Five factor markers [41,42]). All the items consisted of a 5-point Likert-type response scale (1 = *very inaccurate*, 5 = *very accurate*), and Cronbach’s α of each personality were reported as 0.92 (neuroticism), 0.86 (conscientiousness), and 0.88 (agreeableness). The instructions for the given survey asked respondents to describe current themselves, not their wish. Example items are: “Get stressed out easily” (neuroticism), “Am always prepared” (conscientiousness), and “Am interested in people” (agreeableness).
- Workaholism. Workaholism was measured by the multidimensional workaholism scale (MWS [43]). There were four subdimensions, which consisted of four items respectively, so there were 16 items in total. Those subdimensions are motivational factors (e.g., “I always have an inner pressure inside of me that drives me to work”), cognitive factors (e.g., “I feel like I cannot stop myself from thinking about working”), emotional factors (e.g., “I feel upset if I have to miss a day of work for any reason”), and behavioral factors (e.g., “I work more than what is expected of me”). The average intercorrelation among those factors was $\underset{r}{¯}=0.61$, which was similar to the results ($\underset{r}{¯}=0.58$) in the study of Clark and colleagues [43]. Additionally, Cronbach’s α of overall workaholism was reported 0.94, and the lowest value of Cronbach’s α was found for emotional factor, which was 0.86. In the present study, the items were rated on 1 to 5 scale (1 = *strongly disagree*, 5 = *strongly agree*).

#### 2.2.6. Demographic Variables

According to the previous research findings, it was found that several demographic variables influenced both participation in telework and the effectiveness of telework [5,7]. Specifically, gender, age, education level, job, and parenthood were identified as potential factors that could influence the process of telework [5]. Accordingly, the present study measured these demographics and checked whether these factors can influence the hypothesized models.

### 2.3. Analytic Approach

Multilevel modeling was conducted to analyze the within-person nested dataset. Specifically, telework, telepressure, and work–life conflict were first put into the model as level 1 variables (i.e., within-person level variables), while the individual difference variables (e.g., conscientiousness, agreeableness, neuroticism, and workaholism) were then put into the model as level 2 variables (i.e., between-person level variables). According to the estimation of the variance of level 1 variables, intraclass correlation coefficients, ICC (1), were 0.79 for telework, 0.66 for telepressure, and 0.76 for work–life conflict, in which it can be interpreted that multilevel modeling was an appropriate approach to explain the hypothesized relationship between these variables. To test the research hypotheses, multiple regression analysis in multilevel modeling was conducted; especially for testing the moderation effects, the within-personal level interaction was estimated through a separate model. This is because all the moderators (e.g., spontaneity of telework, regularity of telework, and telepressure) in this study were assumed to be within-person level variables. All the analyses were performed with the “lme4” package in the open-source software R.

Before testing the hypotheses in this study, multilevel confirmatory factor analysis (MCFA) for Level 1 variables was run to test measurement invariance between the level 1 and 2 models. The hypothesized model was a three-factor model consisting of telepressure, work-to-life conflict, and life-to-work conflict at both levels 1 and 2. Although telework is also a within-personal level variable, it was excluded in this analysis because it had a different response format. The model fit indices of the hypothesized model were acceptable: $x^{2}(264)=1010.345,\;p\;<\;0.001,\;\mathrm{CFI}=0.922,\;\mathrm{RMSEA}=0.056,\;\mathrm{SRMR}=0.053$. Additionally, because of model comparison, it was found that the hypothesized three-factor model was more suitable for explaining the given data than the model assuming a single-factor model only at levels 1 or 2, as well as the model assuming a single-factor model at both levels. Among the three competitive models, the model assuming a single factor construct only at level 2 showed acceptable fit indices: $x^{2}(267)=1658.608,\;p\;<\;0.001,\;\mathrm{CFI}=0.855,$ RMSEA=0.076, SRMR=0.124. However, the hypothesized three-factor model reported significantly better fit: $\Delta x^{2}(3)=648.263,\;p\;<\;0.001$. Therefore, it was confirmed that the factor model for the within-person level variables was applied equally at both levels 1 and 2. In addition to the MCFA for within-person level variables, since this study includes four between-person level variables, confirmatory factor analysis (CFA) for these variables was also conducted. Since the between-person level variables were measured only in the first survey, the CFA was performed based on only the data obtained in the first survey. Considering the relatively large number of items, item parcels were made using the factorial algorithm suggested by [44]. In this method, items are assigned to each parcel in a balanced way, based on the factor loadings. With the derived item parcels, it was found that the four-factor model for between-person level variables fit the current data, $x^{2}(59)=170.647,\;p\;<\;0.001,\;\mathrm{CFI}=0.960,\;\mathrm{RMSEA}=0.076,\;\mathrm{SRMR}=0.071$. Additionally, this four-factor model showed a better fit, compared to the single-factor model, $\Delta x^{2}(6)=1764.335,\;p\;<\;0.001$. To sum up, the results from MCFA for level 1 variables and CFA for level 2 variables supported the construct validity of the hypothesized models in the current data.

For control variables, the influence of demographic variables on the research model was estimated through both the correlation analysis between continuous demographic variables (e.g., age and the number of children), research variables (e.g., telework, telepressure, and work–life conflict), and the analysis of variance (ANOVA) for categorical demographic variables (e.g., gender, job, and education level). As a result, only age was found to have a significant relationship with telepressure and work–life conflict, and the other demographic variables did not show significant relationships with the research variables used in this study. Specifically, it was found that the younger the age, the higher telepressure (r=−0.18, p < 0.01) and work–life conflict (r=−0.20, p < 0.01). Accordingly, in this study, not all demographic variables were used, but only age variable was put into the research model as a control variable. In addition to the demographic variables, when examining the interaction effect of telework and telepressure on work–life conflict, there is one more consideration. We need to verify whether telepressure has a unique explanatory power beyond various individual difference variables. Grawitch and colleagues [35] pointed out that telepressure did not show a unique explanatory variance beyond individual difference variables, such as workaholism, neuroticism, and conscientiousness, in predicting several well-being indicators (e.g., emotional exhaustion, psychological detachment, and satisfaction with work–life balance) and asked for further exploration of the specific role of telepressure. Thus, the current study examined whether telepressure and the interaction of telework and telepressure can predict work–life conflict after controlling other individual difference variables linked to telepressure. Similar to the study of Grawitch and colleagues [35], the individual difference variables (e.g., workaholism, neuroticism, and conscientiousness) were once again controlled; in the current study, agreeableness was additionally controlled. All the individual difference variables here, except agreeableness, were controlled because they were reported as predictors of telepressure in previous studies [35], and it was expected that the effect of individual differences on part in telepressure would be excluded, to some extent, through this process. In terms of agreeableness, since it is a personality trait that involves altruism, nurturance, caring, and emotional support [45,46], employees who have high agreeableness tend to engage in impression management to avoid losing favor from others [47]. Regarding impression management, Barber and Santuzzi [10] claimed that telepressure can be interpreted as a cognitive aspect of impression management in that employees may feel more telepressure to prevent negative impressions from supervisors or coworkers. To sum up, people who have high agreeableness might try to avoid losing favor from others by replying quickly, and it might lead to high telepressure. Thus, agreeableness was controlled along with other variables because it could be related to telepressure.

## 3. Results

### 3.1. Preliminary Analysis

The correlation coefficients, means, standard deviations, and reliability estimates of the study variables were presented in Table 1. According to the correlation analysis, telepressure was positively associated with work–life conflict (r=0.12, p < 0.01). Additionally, the correlation between telework and work–life conflict was weak and non-significant (r=−0.07, p=0.073), which was expected, given the mixed findings of previous studies on the relationship between these two.

### 3.2. Hypothesis Testing

First, this study investigated the role of the qualitative aspects of telework, when it comes to explaining the relationship between telework and work–life conflict. According to the analysis, all the moderation effects of the qualitative attributes that this study measured were not statistically significant. Therefore, Hypotheses 1 and 2 were not supported. The analysis results were presented in Table 2.

Next, this study investigated whether telework has a differently impact on different types of work–life conflict: work-to-life conflict (WTLC) and life-to-work conflict (LTWC). According to the analysis results in Table 3, the effect of telework on reducing WTLC was found to be significant (γ=−0.38, β=−0.111, p < 0.01), but the relationship between telework and LTWC was not statistically significant (γ=−0.05, β=−0.018, p=0.725). Thus, Hypotheses 3-1 and 3-2 were supported. For more statistically meaningful comparisons, the standardized coefficients for each model were also estimated, and the regression lines of telework on WTLC and LTWC were found to be different.

**Figure 2:**
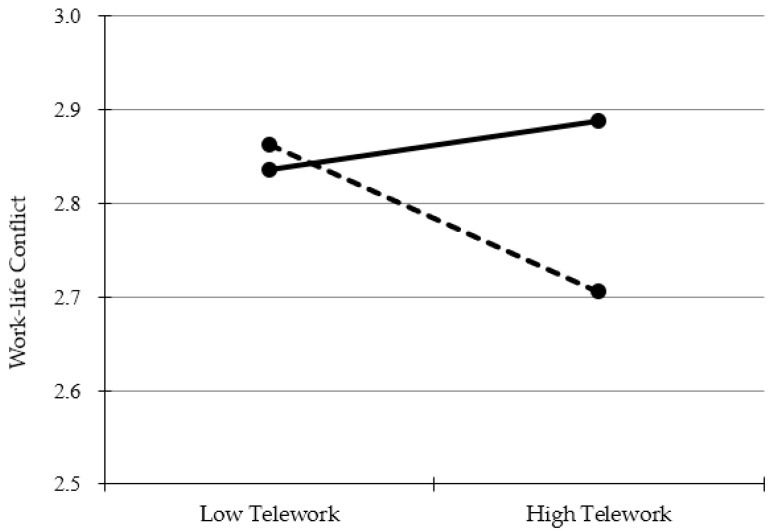
Interaction effect of telework and telepressure on work–life conflict.

Table 4 presents the moderating effect of telepressure. Since all the variables in the moderation analysis were within-person level variables, the path coefficients at level 1 were calculated. It was found that, although telework was only marginally associated with work–life conflict (γ=−0.23, β=−0.08, p=0.060), the interaction effect between telework and telepressure on work–life conflict was statistically significant (γ=1.01, β=0.36, p < 0.05). Therefore, Hypothesis 4 was supported. To explain this interaction effect, it has been shown that telework tends to alleviate work–life conflict for those experiencing low telepressure; conversely, telework seems to have no relationship with work–life conflict or it even tends to increase work–life conflict for those experiencing high telepressure. The visualized interaction effect can be found in Figure 2. Figure 2 was created by dividing entire data into a case greater by 1 standard deviation from the average values and a case smaller by 1 standard deviation after calculating the average and standard deviation of the predictor and moderator.

In addition to testing Hypothesis 4, we added four individual difference variables in the research model, which tests the interaction effect between telework and telepressure on work–life conflict. Even though all the individual difference variables were significantly related to work–life conflict, telepressure was still statistically significant (γ=0.09, β=0.10, p < 0.01), and the interaction term was also marginally significant (γ=0.89, β=0.32, p=0.051). Therefore, it can be argued that telepressure and its moderation effect have a unique explanatory variance in work–life conflict beyond the influence of individual differences. The unstandardized coefficients were presented in Table 5.

### 3.3. Supplementary Analysis

#### 3.3.1. Moderation Effects of Telepressure on WTLC and LTWC

Since the current study found that the relationship between telework and work–life conflict differs depending on the direction of work–life conflict, it was also tested whether the moderation effects of telepressure on work–life conflict show a similar pattern. According to the analysis, the interaction of telework and telepressure was statistically significant only when predicting WTLC (γ=1.35, β=0.39, p < 0.05). Like telework, the moderating role of telepressure did not show a statistically significant effect on LTWC (γ=0.64, β=0.23, p=0.242).

#### 3.3.2. Cross-Level Interaction

Although this study tested the within-person level interaction effect between telework and telepressure on work–life conflict, it is possible to consider cross-level interaction effects. Of course, previous researchers have claimed that telepressure is not an individual difference variable; however, considering the small within-person level variance of telepressure, one could argue that telepressure might be an individual difference variable that was relatively stable over time. To test this possibility, we centered telepressure with grand mean centering, such as other between-person level variables, and tested the cross-level interaction effect between telework and telepressure as supplementary analysis. According to the analysis, however, the interaction effect was not statistically significant (γ=0.11, β=0.03, p=0.507). This result does not mean that telepressure is not an individual difference variable, but it can be interpreted that at least the moderating effects of telepressure appeared differently at different survey periods in this study.

## 4. Discussion

The main purpose of this study was to test moderators of the relationship between telework and work–life conflict. First, we explored the potential impacts of the qualitative aspects of telework, but neither moderated the relationship between telework and work–life conflict. However, it should be noted that, in the current study, there were far more people who voluntarily and regularly participated in telework than those who did not. Since this disproportionate distribution may have affected the analysis results, it would be helpful to retest the research hypotheses using other samples or research methods. In addition, since this study included responses of neutral opinions on voluntariness and regularity, data from people who responded that their telework was neither voluntary (or regular) nor involuntary (or irregular) were included in the analysis. Thus, it may be useful to utilize a binary question format excluding neutral response option and adopt a new analysis method based on a t-test or analysis of variance (ANOVA).

Second, we identified the role of telepressure in explaining the inconsistent relationship between telework and work–life conflict. It was found that telepressure moderated this relationship; specifically, high levels of telepressure weakened the negative relationship between telework and work–life conflict. In other words, telework could reduce work–life conflict for those with low telepressure, but this effect became weaker as telepressure increased. Moreover, this moderation effect was still marginally statistically significant, even after individual difference variables (e.g., workaholism, neuroticism, conscientiousness, and agreeableness) were controlled. Therefore, it can be argued that the role of telepressure can be distinguished from those individual differences that may lead to telepressure, which explains the unique variance in work–life conflict.

Third, the present study explored whether the direction of work–life conflict could explain the inconsistent impact of telework. Indeed, the positive effect of telework on decreasing work–life conflict was found for the case where work role intervenes life role (i.e., work-to-life conflict; WTLC) but not for the case where life role intervenes work (i.e., life-to-work conflict; LTWC). These research findings are consistent with the proposals and results from previous conceptual and empirical work [6,9]. The finding that the relationship between telework and LTWC was not significant may mean that the relationship between the two is more complicated than that of telework and WTLC. For instance, Mesmer-Magnus and Viswesvaran [49] reported that non-work stressors correlate more with family-to-work conflict than with work-to-family conflict. Applying their findings to telework and work–life conflict, since those working at home can be more frequently exposed to non-work stressors [9], LTWC may be impacted by telework. In other words, it suggests that, to investigate the relationship between telework and overall work–life conflict (i.e., both WTLC and LTWC), it is necessary to investigate the overall influence of the non-work domain, as well as the work domain or task itself.

### 4.1. Theoretical Implications

The present study has several important theoretical implications. First, the results of this study suggest some plausible explanations for the inconsistent effects of telework. Given that many organizations have already implemented telework, this is an urgent research gap. Through the present study, we tested the possibility of some boundary conditions in the relationship between telework and work–life conflict. As a result, it was found that the relationship between telework and work–life conflict changed, depending on the level of telepressure. Therefore, it can be argued that variability in the level of telepressure is one of the reasons for differing relationships between telework and work–life conflict in previous studies. Moreover, although the possibility that telepressure is an individual difference variable, rather than one determined by the external environment, as was previously suggested [35], according to the analysis of this study, it is also unlikely that telepressure is determined only by individual differences. In the process of examining the moderating effect of telepressure, the within-person level interaction was found to be significant, but the cross-level interaction was not significant, suggesting that the moderating effect of telepressure might be different for each time point, measured at one-month intervals in this study.

Second, the current study suggests that the distinction between work-to-life conflict (WTLC) and life-to-work conflict (LTWC) in telework research is an important consideration. In general, one of the reasons why the mixed relationship between telework and work–life conflict is problematic may be that there is an expectation that telework mitigates general work–life conflict. However, does work–life conflict here include both WTLC and LTWC? Many people will agree that work flexibility through telework can help alleviate WTLC, but only a few studies have discussed how telework can affect LTWC [6,9]. Like those previous studies, the present study found that telework could alleviate WTLC, but could not decrease LTWC. Moreover, the supplementary analysis found that the interaction effect of telework and telepressure was also significant only for WTLC, not LTWC. These findings suggest that the direction of work–life conflict is an important consideration when discussing the relationship between telework and work–life conflict. Then, how should we approach the relationship between telework and work–life conflict in the future? Excluding the discussion about LTWC, when describing the role of telework, can be one way. However, considering that work–life conflict, as it is currently defined in the literature, includes both WTLC and LTWC; a deeper exploration of the relationship between telework and LTWC will be of great help to future research. For example, information regarding potential non-work stressors, such as whether an individual lives with other people or their workspace is separated in the house, can help researchers better understand the relationship between telework and LTWC.

### 4.2. Practical Implications

The present study also provides some important implications for practice. In this present study, it was found that the participant’s telepressure impacts the effectiveness of telework. Therefore, it is recommended that organizations consider their employees’ telepressure before implementing telework policies. Since telepressure is not a trait that remains unchanged, similar to personality, organizations may be able to lower it through various policy changes [10]. For example, Barber and Santuzzi [10] suggested that telepressure can be reduced by setting specific guidelines regarding the expected response time for work-related communication or training employees on how to restrain themselves when they feel telepressure. Until these steps to reduce individuals’ telepressure are implemented, it is doubtful that telework will decrease work–life conflict.

Second, this study found that telework is helpful for reducing work-to-life conflict (WTLC), but not life-to-work conflict (LTWC). This finding suggests that, in order to reduce the overall work–life conflict through telework, the greatest consideration should go to the negative influence of employees’ roles in the non-work domain on their roles in the work domain. To do this, it may first be necessary to inform employees that telework may not help LTWC. Only when there is an awareness of this problem situation can a solution be developed. Of course, even if the problem is recognized, support for reducing LTWC at the organizational level may be more difficult than support for reducing WTLC. Additionally, since LTWC could be seen more as the responsibility of individual employees, since most of the sources of LTWC are not from workplace, organizations may feel less responsibility to intervene to decrease LTWC [50]. However, when considering the effectiveness of telework from an organization’s point of view, LTWC does impact several outcomes, including employee productivity; we argue that support for mitigating employees’ LTWC is needed at the organizational level, in order to maximize the effectiveness of telework. One potential solution would be to train supervisors to be positive role models, with respect to balancing work and nonwork domains [51]. As such, given that direct organizational interventions designed to impact employees’ personal lives are still controversial [50], it might be more appropriate to take a more indirect approach, such as creating a family supportive climate [52].

### 4.3. Limitations and Future Research Directions

Although this study provides some useful explanations for the inconsistent effects of telework, there are also some limitations. First, it is difficult to estimate the exact causation with the method used in this study. Although a within-person nested dataset was obtained through repeated measurements over consistent time intervals, no time precedence was applied between the predictor and outcome variables. Of course, as more organizations implement telework policies, due to the COVID-19, it is more likely that telework affects work–life conflict. However, if a time lag can be provided between variables through experience sampling methodology (e.g., daily diary technique) in a follow-up study, it will be possible to estimate the causation more accurately between telework and work–life conflict.

Second, the within-person level variations of the level 1 variables were quite small. Although the value of ICC (1) suggests the appropriateness of multilevel analysis for the data, participants in this study did not experience significant changes in telework, telepressure, and work–life conflict during the three surveys. However, given that this study found a significant moderating effect of telepressure, despite this small variance, this suggests that our finding was robust. Nevertheless, if future studies want to investigate the dynamics between the variables studied in this study, it would be helpful to adopt longer time intervals or conduct experimental studies that can manipulate the implementation of telework to ensure sufficient variation at the within-person level.

Third, although several demographic variables were measured and age was controlled in all the analyses, there is still a potential problem of unobserved variables. For example, external variables, such as specific residence, state policies, homeschooling of children, or whether an individual has a proper environment for working at home, could influence the effect of telework, but these were not measured in the present study. The concept of regularity of telework might include this element, in terms of consistency, but the current study might not have captured the overall characteristics of telework environments because we focused on the frequency of telework in the process of operationalizing the regularity of telework. Considering that the purpose of the present study was to explore factors that may affect the inconsistent effects of telework, other external variables, including the working environment, need to be considered when replicating or extending this study.

Fourth, as one of the reviewers pointed out, the present study reported high Cronbach Alpha values over 0.90 for some research variables. Extremely high Cronbach alphas might be a sign of the redundancy of items; indeed, Streiner [53] suggested 0.90 as a maximum alpha value. However, the scales that reported high alpha values in the present study have already been utilized by many previous studies, and our alphas were very similar to the alpha values reported by the initial studies responsible for developing the scales [10,32,41,42,43]. Additionally, since the redundancy of items might harm the construct validity, it was tested via factory analysis. Specifically, the convergent validity within the factors (i.e., each variable) and discriminant validity between factors were both supported by conducting multilevel confirmatory factor analysis (MCFA) for within-person level variables and confirmatory factor analysis (CFA) for between-person level variables.

Fifth, since the present study relied on data from only one source (e.g., Prolific), it is unclear whether the sample obtained in this study was representative of the entire population of working adults. Comparing the demographic variables (e.g., age, gender, job, education level, and the number of children) measured in the present study to the general U.S. population, our sample seems to be younger and have a higher education level, but there was not much difference, compared to samples used in other telework studies [9,12,37]. However, regarding the number of children, the sample used in the present study and other telework studies were different. In many telework studies, most participants reported having more than one child, whereas, in the present study, 204 (62.2%) out of 328 participants reported that they have no children. This may be a limitation of the present study; on the other hand, considering that the number of single-person households is increasing rapidly [22,23], it is still useful to understand the inconsistent effects of telework on single-person households.

Sixth, although research using crowdworking platforms, such as Amazon MTurk or Prolific, is increasing [54], the use of these platforms is not free from limitations. Even though the quality of Prolific data may be expected to be superior to other crowdworking platforms, in terms of transparency [55], this strength does not address all the problems that the crowdworking platforms have. For example, considering that Prolific members can be more familiar with the survey process, a heuristic providing a similar response to a relatively similar question may occur more for those who are familiar with the survey than those who are not. To minimize such potential problems, the best practices for using a crowdworking platform, suggested by Aguinis and colleagues [54], were utilized in an effort to minimize the problems. First, the inclusion criteria (e.g., (1) currently residing in the United States, (2) working full-time, and (3) performing telework at least once a week) were applied to secure the samples that share similar characteristics with the population set in the current study. Next, several questions to detect careless responses, which have been frequently found in online surveys, were included in the questionnaire. However, there were no participants who provided careless responses, according to the data analysis. The example item was: “It’s important that you pay attention to this study. Please tick ‘Strongly Agree’”.

Lastly, since our data were collected during the COVID-19 pandemic, it may be difficult to generalize the results to future time periods. For example, compared to past studies, where the proportion of telework hours to the total work hours was relatively small [9,37], higher proportion values were reported in the current study. Almost 75% of the total cases reported that telework occupies more than half of the total work, and 60% among them reported that all their tasks were performed through telework. Although this did not impact the analysis itself, because the normality of the residuals was confirmed in the research model, research findings based on such extremely negatively skewed data may be difficult to generalize. On one hand, however, as COVID-19 is a global event, it may be more inaccurate to apply the results of the past studies before the COVID-19 pandemic to the present. Many experts predict that the COVID-19 crisis has forced many organizations to implement telework policy, and this once-initiated wave of telework will be maintained, to some extent, even after the COVID-19 crisis is over [3,4,5]. If so, should various research findings from the COVID-19 era be viewed as special cases influenced by the COVID-19 pandemic? Or should we see it as a new paradigm that will be maintained, even after the end of the COVID-19 era? To answer these questions, the replication and review of telework research findings is required more than ever.

## 5. Conclusions

This study provides empirical evidence that the inconsistent findings, regarding the relationship between telework and work–life conflicts, may be due to the direction of work–life conflict and level of telepressure. Specifically, it was found that the impacts of telework differed between work-to-life conflict (WTLC) and life-to-work conflict (LTWC). Especially since the relationship between telework and LTWC was not statistically significant, we argue that consideration of LTWC should be preceded when examining the relationship between telework and work–life conflict. Next, the moderation effect of telepressure was statistically significant, suggesting that the effect of telework reducing work–life conflict is weakened for people with high telepressure. Therefore, we argue that telepressure should be considered when investigating the effect of telework and implementing organizational telework policies. At the organizational level, organizational policies setting guidelines related to work-related communication and employee training on how to cope with telepressure can be useful to reduce employees’ telepressure and ultimately secure the positive effect of telework on work–life conflict.

## Figures and Tables

**Table 1:** Means, standard errors, reliability estimates, and correlations between study variables.

	1	2	3	4	5	6	7
1. Telework ^a^		0.02	−0.07 ^†^				
2. Telepressure ^a^	−0.02		0.12 **				
3. Work–life conflict ^a^	−0.12 *	0.16 *					
4. Conscientiousness ^b^	−0.05	−0.03	−0.38 ***				
5. Agreeableness ^b^	−0.07	0.12 *	−0.16 **	0.20 ***			
6. Neuroticism ^b^	0.06	0.19 **	0.46 ***	−0.41 ***	−0.10 ^†^		
7. Workaholism ^b^	−0.19 ***	0.19 **	0.36 ***	0.10 ^†^	0.11 *	0.05	
M	0.73	3.48	2.31	3.85	4.00	2.66	2.68
Within-person SD	0.01	0.20	0.11	-	-	-	-
Between-person SD	0.24	2.06	1.72	0.67	0.67	0.94	0.85
Cronbach’s alpha	-	0.93 ^c^	0.93 ^c^	0.86	0.88	0.92	0.94
Omega	-	0.84	0.93	-	-	-	-

*Note.* Correlations below the diagonal represent between-person correlations (n=328). Correlations above the diagonal represent within-person correlations (n=893). To calculate between-person correlations, average scores of within-person variables across times were utilized. ^a^ Within-person variables. ^b^ Between-person variables. ^c^ Average Cronbach’s alpha across times. Omega values are reported as within-person level reliability estimates [48]. ^†^ *p* < 0.1, * *p* < 0.05, ** *p* < 0.01, *** *p* < 0.001.

**Table 2:** Moderation effects of qualitative aspects of telework on work–life conflict.

	Work–Life Conflict		
Variable	Estimate	β	*SE*
Intercept	2.31 ***	0.01	0.043
Age ^b^	−0.01 ***	−0.17 ***	0.004
Telework ^a^	−0.20	−0.07	0.122
Spontaneity ^a^	−0.03	−0.04	0.021
Telework × spontaneity	0.19	0.08	0.188
Intercept	2.31 ***	0.01	0.043
Age ^b^	−0.01 ***	−0.17 ***	0.004
Telework ^a^	−0.17	−0.06	0.124
Regularity ^a^	−0.02	−0.03	0.025
Telework × regularity	0.23	0.09	0.180

*Note*. Unstandardized coefficient values in each model were estimated independently. “*β*” represents the standardized coefficient value. ^a^ Within-person variables. ^b^ Between-person variables. *** *p* < 0.001.

**Table 3:** Comparison depending on the direction of work–life conflict.

	Work-to-Life Conflict (WTLC)			Life-to-Work Conflict (LTWC)		
Variable	Estimate (*γ*)	β	*SE*	Estimate (*γ*)	β	*SE*
Intercept	2.53 ***	0.012	0.055	2.10 ***	0.002	0.043
Age ^b^	−0.02 **	−0.150 **	0.005	−0.01 **	−0.148 **	0.004
Telework ^a^	−0.38 **	−0.111 **	0.141	−0.05	−0.018	0.144

*Note*. All the coefficient values in each model were estimated independently. “*β*” represents the standardized coefficient value. ^a^ Within-person variables. ^b^ Between-person variables. ** *p* < 0.01, *** *p* < 0.001.

**Table 4:** Moderation effect of telepressure on work–life conflict.

	Work–Life Conflict		
Variable	Estimate	β	*SE*
Intercept	2.31 ***	0.01	0.043
Age ^b^	−0.01 ***	−0.17 ***	0.004
Telework ^a^	−0.23 ^†^	−0.08 ^†^	0.120
Telepressure ^a^	0.09 **	0.10 **	0.031
Telework × telepressure	1.01 *	0.36 *	0.467

*Note*. Two models were estimated: a model that assumes telepressure as between-person level variable and a model that assumes telepressure as within-person level variable. ^a^ Within-person variables. ^b^ Between-person variables. ^†^ *p* < 0.1, * *p* < 0.05, ** *p* < 0.01, *** *p* < 0.001.

**Table 5:** Multilevel multiple regression results predicting work–life conflict.

	Work–Life Conflict		
Variable	Estimate	β	*SE*
Intercept	2.31 ***	0.01	0.035
Age ^b^	−0.00	−0.04	0.003
Telework ^a^	−0.23 ^†^	−0.08 ^†^	0.120
Workaholism ^b^	0.34 ***	0.33 ***	0.043
Neuroticism ^b^	0.25 ***	0.27 ***	0.042
Conscientiousness ^b^	−0.29 ***	−0.22 ***	0.060
Agreeableness ^b^	−0.13 *	−0.10 *	0.054
Telepressure ^a^	0.09 **	0.10 **	0.031
Telework × telepressure	0.89 ^†^	0.32 ^†^	0.455

^a^ Within-person variables. ^b^ Between-person variables. ^†^ *p* < 0.1, * *p* < 0.05, ** *p* < 0.01, *** *p* < 0.001.

## Data Availability

Not applicable.
